# Impoverished Inhibitory Control Exacerbates Multisensory Impairments in Older Fallers

**DOI:** 10.3389/fnagi.2021.700787

**Published:** 2021-09-24

**Authors:** Alexandra N. Scurry, Zachary Lovelady, Daniela M. Lemus, Fang Jiang

**Affiliations:** Department of Psychology, University of Nevada, Reno, Reno, NV, United States

**Keywords:** inhibition, multisensory processing, sound-induced flash illusion, fall-risk, aging

## Abstract

Impaired temporal perception of multisensory cues is a common phenomenon observed in older adults that can lead to unreliable percepts of the external world. For instance, the sound induced flash illusion (SIFI) can induce an illusory percept of a second flash by presenting a beep close in time to an initial flash-beep pair. Older adults that have enhanced susceptibility to a fall demonstrate significantly stronger illusion percepts during the SIFI task compared to those older adults without any history of falling. We hypothesize that a global inhibitory deficit may be driving the impairments across both postural stability and multisensory function in older adults with a fall history (FH). We investigated oscillatory activity and perceptual performance during the SIFI task, to understand how active sensory processing, measured by gamma (30–80 Hz) power, was regulated by alpha activity (8–13 Hz), oscillations that reflect inhibitory control. Compared to young adults (YA), the FH and non-faller (NF) groups demonstrated enhanced susceptibility to the SIFI. Further, the FH group had significantly greater illusion strength compared to the NF group. The FH group also showed significantly impaired performance relative to YA during congruent trials (2 flash-beep pairs resulting in veridical perception of 2 flashes). In illusion compared to non-illusion trials, the NF group demonstrated reduced alpha power (or diminished inhibitory control). Relative to YA and NF, the FH group showed reduced phase-amplitude coupling between alpha and gamma activity in non-illusion trials. This loss of inhibitory capacity over sensory processing in FH compared to NF suggests a more severe change than that consequent of natural aging.

## Introduction

An individual’s experience of the natural world is largely dictated by innate biases and sensitivities toward external stimuli. For instance, as light is propagated at a rapidly faster speed relative to sound, the brain learns this relationship over development and becomes extremely sensitive to auditory-leading signals, as compared to visual-leading. This asymmetry has been continuously observed and reported, particularly in the case of the temporal binding window (TBW) (Powers et al., 2009; Stevenson and Wallace, 2013), an estimate that quantifies the likelihood of perceptually binding two stimuli that are separated by variable temporal delays. Learned temporal relationships of naturally occurring stimuli drive the flexibility of this window (Murray et al., 2016). For instance, TBWs estimated using simple audiovisual stimuli (e.g., a flash and a beep) are much narrower compared to TBWs derived from using more complex audiovisual stimuli (e.g., the visual and auditory cues from speech) (Van Atteveldt et al., 2007; Stevenson and Wallace, 2013). The broader TBW observed in this more ethologically relevant context is not surprising. The brain requires additional flexibility, or increased processing time, to decode the unitary signals and perceive them as either a single, coherent source or as two separate and distinct sources. The temporal relationship between the constituent unimodal parts of a multisensory event is the major driver in dictating this coherent versus segregated perception. Reductions in sensitivity toward audiovisual temporal differences would therefore result in incoherent perceptions and difficulties in experiencing and navigating the environment.

In the healthy older adult, changes in temporal thresholds do occur as a natural consequence of the aging process and thus, deficits in unisensory and multisensory processing are observed (Allison et al., 1984; Schmolesky et al., 2000; Stephen et al., 2010; Ng and Recanzone, 2018). However, explicitly controlling for differences in unisensory processing thresholds cannot completely explain multisensory impairments reported in older adults (Chan et al., 2014). Therefore, additional age-related cortical modifications that affect multisensory-specific functionality are likely at fault. In the auditory region of senescent rhesus macaques, neurons displayed increased spontaneous activity, reduced selectivity in coding patterns and broader-tuned neurons relative to young controls (Ng and Recanzone, 2018). Similar age-related alterations were observed in visual cortex of older macaques with increased responsiveness and broader-tuning curves toward the orientation and direction of visual stimuli (Schmolesky et al., 2000). In addition, the proportion of audiovisual neurons exhibiting tuning toward low spatial frequencies was reduced in the aged rat model while those audiovisual neurons exhibiting band-pass tuning was increased suggesting compensation for this reduction in sensitivity (Costa et al., 2016). In both multisensory and primary sensory regions, the aged cortex displays heightened excitability accompanied by broader-tuning neuronal profiles responsible for impaired precision and reduced reliability in sensory processing. This suggests diminished signal to noise and a loss of inhibitory control on the processing and decoding strategies necessary for precise representations of sensory inputs. In fact, visual cortex neurons of older macaques that were treated with a GABA agonist demonstrated recovered response profiles similar to young monkeys (Leventhal et al., 2003).

The loss of inhibition hypothesis supported by these findings is a feasible explanation for the impaired multisensory processing reported in older adults. While performing an auditory target detection task, older adults demonstrated altered evoked responses consistent with reduced top-down inhibition via frontal areas (Stothart and Kazanina, 2016). In addition, when incongruent visual information was presented with auditory, older adults demonstrated less efficient and more distributed cortical processing to retain perceptual constancy (Stothart and Kazanina, 2016). Older adults also have reduced ability to ignore irrelevant, distracting information, likely due to the reduced signal to noise present in the aging brain (Tun et al., 2002; Van Gerven and Guerreiro, 2016). Indeed, older adults demonstrated poor suppression of visually distracting, task-irrelevant information in a working memory task (Borghini et al., 2018). However, the ability to filter irrelevant visual cues was recovered by the application of alpha *trans-*alternating current stimulation over the parietal lobe indicating that reduced power in the alpha band is related to the poor inhibitory control found in the aging population (Borghini et al., 2018). This is not a surprising finding given the known functional role of top-down inhibitory control reflected by cortical oscillatory activity in the alpha range (7–13 Hz) (Klimesch et al., 2007).

Intact, coordinated alpha activity provides dynamic and specific control of processing in other areas of the brain by (1) controlling the amount of cognitive resources applied to the task at hand and (2) providing discrete temporal windows for processing to occur (Klimesch et al., 2007; Jensen and Mazaheri, 2010; Klimesch, 2012). For instance, feed-forward processing of sensory information is modulated by the phase of alpha activity so that bursts of activity occur during the troughs of the alpha cycle while suppression of active processing occurs at alpha peaks (Bonnefond and Jensen, 2015). Indeed, this sensory gating via inhibition during multisensory tasks is necessary to filter irrelevant sensory information and promote processing in relevant areas (Jensen and Mazaheri, 2010; Keller et al., 2017). Therefore, proper inhibitory control via alpha activity enables robust synchronization of activity between cortical regions most relevant for the present, required function.

Synchronization of oscillations across cortical regions is particularly relevant for multisensory processing as it enables the transfer and integration of information from multiple modalities. One mechanism involved in cross-modal influence is phase resetting where congruent audiovisual signals induce stronger phase coherence and results in faster behavioral response times to a congruent multisensory stimulus compared to an incongruent multisensory cue or single unisensory cues (Keil and Senkowski, 2018). Furthermore, congruent visual-tactile motion as well as temporally aligned audiovisual stimuli induced stronger gamma band (30–70 Hz) power within the respective primary sensory areas, indicative of enhanced low-level processing that is synchronized across these cortical regions (Senkowski et al., 2007; Krebber et al., 2015). These findings are in line with the known function of gamma oscillations in the active, bottom-up processing of low-level inputs.

In addition to enhanced and synchronized gamma power, the involvement of alpha activity regulating this feed-forward processing is also an integral mechanism. A commonly reported coupling between alpha and gamma demonstrates pulsed gating by inhibition induced by alpha activity wherein gamma power is lowest at peaks in the alpha band and highest at troughs in the alpha band (Jensen et al., 2014; Bonnefond and Jensen, 2015). This phase-amplitude coupling (PAC) has helped to explain and predict the percepts associated with the sound-induced flash illusion (SIFI) task where a simultaneous flash and beep is followed close in time by a secondary beep in order to induce the illusory perception of 2 flashes, also known as a fission or double-flash illusion (Shams et al., 2002, 2000). A significant increase in gamma power was observed for illusory versus non-illusory trials within occipital area suggesting that gamma activity reflects the low-level perceptual binding of multisensory stimuli (Bhattacharya et al., 2002). In addition, stronger alpha power further limits the length of the duty-cycle by which gamma activity can function providing an additional way of modulating lower-level processing (Jensen and Mazaheri, 2010). Decreased alpha power was associated with higher probabilities of experiencing the illusion, likely because stronger alpha activity inhibits the bottom-up processing reflected by the gamma band within the occipital area (Cecere et al., 2015; Keil and Senkowski, 2017).

The SIFI is an especially intriguing multisensory illusion as the same physical stimulus can elicit two opposing perceptual states. Therefore, it is an excellent task to parse out differences in cortical processes that drive opposing percepts. In addition, the stimulus onset asynchrony (SOA), or temporal difference, between the simultaneous flash/beep pair and the second beep can elucidate perceptual differences driven by altered temporal sensitivity, as observed in young versus older adults performing the fission SIFI task (McGovern et al., 2014). Most intriguingly, the SIFI task has also distinguished multisensory perceptual differences between older adults with a history of falling compared to those older adults without. For instance, older adults with a history of falling had significantly worse accuracy in perceiving the single veridical flash (versus the illusory 2 flashes) compared to older non-fallers and young adults at SOAs ≥110 ms indicating that multisensory temporal sensitivity was severely impaired in the fall history group (Setti et al., 2011). In a separate study, older adults with and without a history of falling performed the SIFI task while maintaining their posture. Variability in postural sway was significantly worse in older fallers while they performed illusory trials of the SIFI task but not control, congruent trials (Stapleton et al., 2014). Following a 5-week balance intervention program, improved postural stability measures were positively correlated with reduced susceptibility to the SIFI in the fall-history group only (Merriman et al., 2015). Taken together, these findings implicate shared deficits within multisensory and postural systems that are present in older adults prone to a fall.

By measuring cortical oscillations in the alpha and gamma band recorded during the SIFI task, the present study sought to examine inhibitory function during multisensory processing and contrast this function between young adults, older adults with a fall history (FH) and those older adults without a fall history (non-fallers; NF). Presuming a global impairment of top-down control in older adults, we expected reduced multisensory function in perceptual measures across older adults and more severe deficits in the FH group. In addition, we hypothesized reduced alpha power, increased gamma power and diminished alpha-gamma PAC during illusion conditions as a consequence of natural aging. While we proposed a more severe deficit of inhibitory capacity in the FH group, we expected more robust deficiencies in these measures of oscillatory activity and coupling for this group.

## Materials and Methods

### Participants

24 young adults (24.16±3.86 years, 15 males), 24 older adults without any history of falling (non-fallers; NF) (69.93 ±3.50 years, 10 males) and 16 older adults with a recent fall history (FH) (73.20 ±3.28 years, 5 males) participated in this study. A fall history was identified as the individual experiencing at least 1 fall in the 18 months preceding experimentation with a fall defined as “unintentionally coming to rest on the ground or lower level” (Tinetti et al., 1988). The average age of the NF group was significantly lower than that of the FH group [*t*(38) = −2.96, *p* < 0.05].

All participants were screened for normal hearing using AudioScope 3,a screening audiometer (Welch Allyn, Skaneateles Falls, NY, United States), and were required to have a pure tone threshold lower than 40 (for older adults) or 25 (for younger adults) dB for 1 and 2 kHz Hearing Level (HL) in both ears. Participants were asked to declare any uncorrected visual deficits and were excluded if they presented with any visual problem such as cataracts or glaucoma. Other exclusion criteria included history of neurological disorders or disease, seizure disorder, brain injury and use of antipsychotic medications. To account for any vestibular or musculoskeletal problems that could contribute to an individual’s risk of falling, all participants were further screened for any chronic pain, use of pain medications, recent musculoskeletal injuries, or any vestibular disorders. Finally, older adults were required to score ≥26 on the Montreal Cognitive Assessment (MoCA) to control for any potential cognitive decline.

Participants provided signed informed consent before any experimentation and were financially compensated for their time. The experimental protocol was reviewed and approved by the Institutional Review Board at the University of Nevada, Reno.

### Stimuli and Equipment

Stimuli were generated using MATLAB (MathWorks, Natick, MA, United States) and Psychtoolbox extensions (Brainard, 1997; Pelli, 1997). The visual stimulus was a stationary white circle with a diameter of 3.5° presented on a grey background in the center of the screen. Auditory stimuli were pure tones of 1,000 Hz created in MATLAB and presented binaurally at 70 dB (measured at the auditory source) via a speaker (Fantech HellScream GS 201, Nepal) directly under the center of the display to approximate the same spatial location as the visual signal. Visual and auditory stimuli were delivered through a Display ++ system with a refresh rate of 120 Hz and an AudioFile stimulus processor, respectively (Cambridge Research Systems, Rochester, United Kingdom). For all experiments, participants sat in front of the display 60 cm away from the screen.

### Sound-Induced Flash Illusion (SIFI) Task

**FIGURE 1:**
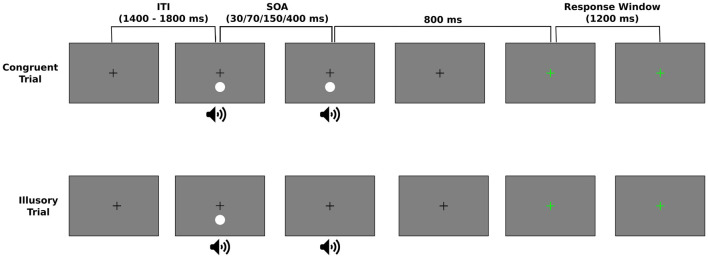
Experimental design of the Sound Induced Flash Illusion (SIFI) task. During a trial, the visual stimulus (white circle) was presented below the fixation cross while the pure tone auditory beep was presented via speakers centered underneath the display. Trials were either a congruent or illusory trial. Congruent trials could either be a single flash-beep pair (1F1B) or two sequential flash-beep pairs (2F2B) that were separated by a variable stimulus onset asynchrony (SOA) (top panel). During the illusory trials, a flash-beep pair was first presented followed by a second auditory beep at some SOA (bottom panel). Regardless of trial type, participants were asked to respond as to how many flashes they perceived during the trial. To prevent motor artifacts, this response was entered 800 ms following the end of the stimulus presentation, indicated by a change in the color of the fixation from black to green. The response window was 1,200 ms and trials were separated by a variably intertrial interval (ITI) between 1,400 and 1,800 ms.

There were 3 possible trial types that could be presented throughout the experiment. In congruent trials, either a flash and beep pair were followed by a second, synchronous flash and beep pair (2F2B) (top panel of Figure 1) or a single flash-beep pair was presented (1F1B). The 1F1B condition was presented on 30 trials. In the 2F2B condition, the second flash-beep pair was delayed by a variable SOA (30, 70, 150, or 400 ms) with each SOA repeated 30 times. In illusory trials, a flash-beep pair was presented simultaneously followed by a second beep (1F2B) (bottom panel of Figure 1). The second beep was delayed by a variable SOA (30, 70, 150, or 400 ms), however, as the temporal limits dictating a person’s susceptibility to the illusion can greatly vary (McGovern et al., 2014) and be influenced by the number of SOAs (Chan et al., 2018), each participant was randomly assigned a single, experimental SOA for the 1F2B condition. There were a total of 240 illusory (1F2B) trials. Table 1 displays the number of participants that performed each of the 4 possible SOA conditions for illusory trials.

**TABLE 1:** Number of participants per group that performed illusory trials for each possible SOA value and were included in the perceptual analysis.

	**30 ms**	**70 ms**	**150 ms**	**400 ms**
**YA**	9 (7/5)	4 (2/2)	5 (3/2)	6
**NF**	5 (5/4)	4 (3/3)	7 (6/5)	8
**FH**	6 (5/5)	6 (4/3)	2 (2/2)	2

*Numbers in parenthesis are the final number of participants in each group included for time-frequency analysis / the final number of participants in each group included for PAC analysis.*

Throughout all experimental trials, a central white fixation was present on the screen. The 17 ms visual stimulus was presented at 4.1° below the fixation (see Figure 1). The auditory stimulus was a 10 ms 1,000 Hz pure tone presented via speakers centered below the display to approximate the spatial location of the visual stimulus. The experimental trials were separated into 8 experimental blocks and the order of trial type and SOAs (for the 2F2B condition) was randomized. Participants were instructed to wait until the fixation cross turned green before they entered their response to reduce contamination from muscle artifacts (see Figure 1). 800 ms following the presentation of the second stimulus in 2F2B or 1F2B trials and of the simultaneous stimulus in 1F1B trials, the fixation turned green for a maximum of a 1,200 ms response window and participants used the number pad on the keyboard to respond as to the number of flashes they perceived during the trial. Trials were separated by an intertrial interval that randomly varied between 1,400 and 1,800 ms.

To ensure participants understood the task and became familiarized with the response procedure and timing, each participant performed a short practice block prior to experimentation that consisted of 9 total trials: 4 illusory, 4 2F2B congruent and 1 1F1B congruent trial. Trial order was randomized, and 2 SOA levels (100 and 500 ms) were used in illusory and 2F2B trials.

### Electroencephalography Acquisition and Pre-processing

Participants performed the SIFI task while EEG data were continuously recorded from a Biosemi 128 Channel system. In addition to the standard 10–20 electrode locations, this system included intermediate positions. Default electrode labels were renamed to approximate the more conventional 10–20 system (see Supplementary Figure 1 in Rossion et al., 2015). 4 additional channels recorded electrooculography (EOG) signals, two channels on the lateral sides of each eye to detect horizontal movement and two channels above and below the right eye to detect vertical movement (e.g., blinks). EEG was sampled at a rate of 512 Hz and processed offline using EEGLAB (v.14_0_0b) and ERPLAB (v.6.1.3) with MATLAB R2013b (MathWorks, Natick, MA, United States).

Due to the altered experimental design in which participants were randomly presented with only one SOA during the illusory 1F2B condition, some participants did not experience any illusion throughout the experiment and their data was excluded for subsequent analysis. Further, across the older adults who received the 400 ms SOA illusory condition, no individual perceived more than 8 illusion trials. Therefore, the 400 ms was completely excluded from further analysis. A total of 12 datasets from YA group, 14 from NF group and 11 from FH group were retained for subsequent EEG analysis.

EEG data were initially bandpass filtered from 0.5 to 125 Hz with a second order, non-causal Butterworth filter and re-referenced to the common average reference. Channels were identified for rejection using the TrimOutlier plugin (v.0.17) based on a threshold of ±200 μV. Across participants, an average of 1.3 (±2.22) channels were rejected and spherically interpolated. Next, epochs of 2,300 ms, beginning 800 ms before trial onset (defined as onset of the first flash-beep pair), were extracted from continuous data. Epochs corrupted by muscle artifacts were identified by visual inspection and an average of 28.58 (±30.58) trials (<5.7%) were rejected across participants. Blink and eye movement artifacts were corrected in the epoched data using Independent Component Analysis (ICA).

### Time-Frequency Analysis

Time-frequency analysis was focused on a region of interest (ROI) based in the occipital area. This ROI was *a priori* selected as it has been implicated for both gamma-mediated processing in the SIFI (Bhattacharya et al., 2002) and alpha activity in occipital sensors has been previously associated with temporal limits of illusory perception (Cecere et al., 2015; Keil and Senkowski, 2017). The occipital ROI was an average of 9 electrodes: PO11, PO1, POO5, POOz, Oz, OIz, POI2, O2, POO6 (McGovern et al., 2014).

Epoched data was categorized into 4 conditions: Illusion trials (illusory 1F2B trials w/2 flash response), non-Illusion trials (illusory 1F2B trials w/1 flash response), 2F2B congruent trials for each of the 4 SOA levels used and 1F1B congruent trials. Table 2 shows the average number of trials retained for time-frequency analysis for each participant group and presented SOA level. Oscillatory activity was analyzed by convolving the EEG data with a set of complex Morlet wavelets. A total of 35, linearly spaced frequencies were used to create the set of 35 wavelets that ranged from 3 to 90 Hz. The full-width at half-maximum (FWHM) ranged from 42.96 to 652.34 ms with increasing wavelet peak frequency. Subsequent analysis extracted power information from 7 to 14 Hz for the alpha band and from 35 to 70 Hz for the gamma band. Raw amplitudes were decibel (dB) normalized using the average power measured from the −500 to −200 ms pre-stimulus window. Table 1 displays the number of datasets used in time-frequency analysis for each participant group and illusory SOA.

**TABLE 2:** Average number of trials (SD) that survived preprocessing for subsequent time-frequency analysis for each group and illusory SOA level.

	**30 ms**	**70 ms**	**150 ms**
**YA**	206.57 (27.31)	229.00 (1.41)	222.67 (10.41)
**NF**	222.20 (8.56)	222.33 (5.03)	224.00 (6.63)
**FH**	222.83 (5.23)	220.75 (5.74)	220 (0.00)

### Phase-Amplitude Coupling (PAC)

A participant’s EEG data was selected for PAC analysis if (1) the participant experienced both conditions (illusion and non-illusion) and (2) that participant had a minimum of 10 trials for each condition. This inclusion criteria resulted in a total of 9 YA, 12 NF, and 10 FH datasets. Table 1 displays the number of datasets for each participant group and illusory SOA used in the PAC analysis. Further, the PAC analysis was focused on a single source, Oz, as this electrode has previously been implicated in coupling between alpha and gamma band (Cecere et al., 2015; Keil and Senkowski, 2017).

Instantaneous phase and amplitude were extracted by convolving the EEG data with complex Morlet wavelets separately for each condition (Samiee and Baillet, 2017; Hirano et al., 2018). A range of linearly spaced frequencies from 7 to 14 Hz and from 34 to 80 Hz were used for phase and amplitude, respectively. To ensure 3 cycles of the lowest phase frequency used, a time window of 36–464 ms, centered around 200 ms post-stimulus (the flash/beep pair), was used within each trial and concatenated across trials. After instantaneous phase and amplitude information was extracted, the PAC values were calculated for each phase-amplitude pair using the following equation:


$$PAC=\left|\frac{1}{n}\sum_{t=1}^{n}a_{t}e^{i\phi_{t}}\right|$$

Where *t* is time point, *a* is power at time point *t*, *i* is the imaginary operator and *ϕ* is the phase angle at time point *t*, and *n* is the total number of timepoints.

Raw PAC values were then converted into z scores using permutation testing. Using the power time series, within each trial, a time point based on the time window of interest was pseudo-randomly chosen as the point to cut the data. This time point was constrained so that at least 45 ms preceded and at most 385 ms followed it. The original second half of the data was then shifted to the front and the original first half of the data was placed after it. A new, permuted PAC value was then computed between this new, shuffled power time series and the original phase time series. A total of 1,500 iterations were performed to create a sampling distribution and a z-score was created using the mean and SE of this sampling distribution.

### Alpha Phase-Locked Power Spectra

In order to differentiate and characterize the results from our initial PAC analysis, we followed an approach from Bonnefond and Jensen (2015) and aligned time-frequency representations of low-frequency gamma band (30–60 Hz) to peaks of 10.5 Hz alpha activity. This particular alpha frequency was chosen as it represented the median frequency in the alpha range showing robust coupling with amplitude of the gamma range (as discovered in the PAC analysis). For each epoch, we convolved the fast Fourier transform of EEG data with that of a complex Morlet wavelet defined by the 10.5 Hz alpha phase frequency. Phase values were extracted from a 300 ms time window centered around 200 ms post-stimulus (the flash/beep pair), this ensured 3 cycles of alpha activity. Separately, power values were estimated using the same approach but with complex Morlet wavelets defined using linearly spaced frequencies from 30 to 60 Hz. Then, peaks of the 10.5 Hz activity were detected by ensuring each peak was surrounded by troughs and corresponding timepoints of the peaks were identified. Time-frequency representations were subsequently re-aligned to be phase-locked with the peak of the cycle. This alignment occurred for epochs that we could extract a full cycle of alpha activity before and after each peak. Power values were then averaged across epochs within the illusion and non-illusion trials for each subject and converted to relative power change with respect to the average activity across the pre-defined time window. These individual time-frequency representations were then used to estimate the alpha phase-locked group average power spectrum of the low-gamma band.

### Perceptual Analysis

To determine the strength of the fission, SIFI illusion, the difference between the accuracy of illusion trials (1F2B) and the accuracy of 1F1B congruent trials was calculated for each individual (Narinesingh et al., 2017). This was done across all participants, including those whose data was excluded for EEG analysis. In addition, accuracy of determining 2 flashes in the 2F2B congruent trials were computed for each SOA value across all participants.

### Statistical Analysis

**FIGURE 2:**
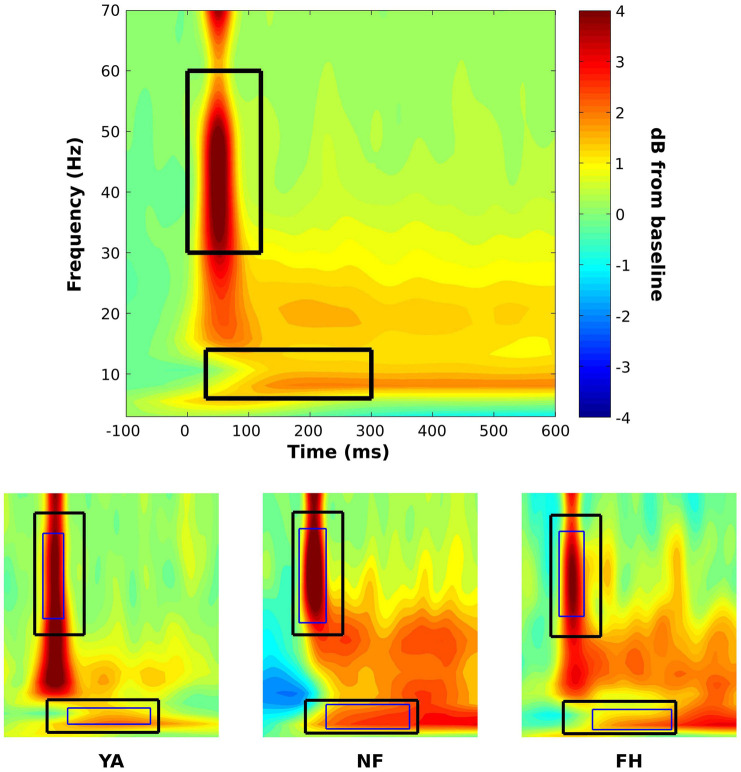
Time-frequency windows for statistical analysis on power spectra. A time-frequency plot that was constructed across groups and conditions (top panel) was visually inspected to identify initial time-frequency windows to extract average alpha (lower box) and gamma power (higher box). Using these pre-defined constraints, subject specific time-frequency windows (bottom panel; thin box within group-defined box) were identified for alpha and gamma using time-frequency plots that were averaged across conditions within each subject. Representative subject-specific time-frequency windows are displayed on time-frequency plots of the respective YA (bottom panel, left plot), NF (bottom panel, middle plot), and FH (bottom panel, right plot) participants. Power values are displayed in dB.

Mixed ANOVAs were performed to determine effects of group and SOA on illusion rate from the SIFI experiment. To determine statistical differences in alpha and gamma power, an initial, group-level time-frequency window was created. Specifically, a time-frequency plot was created by averaging across all subjects and all conditions. This plot was then visually inspected, and 2 separate time-frequency windows were identified to extract gamma and alpha power. At this group level, the temporal boundaries were 0–120 ms and 30–300 ms while the frequency boundaries were 30–60 Hz and 6–14 Hz for the gamma and alpha bands, respectively (Figure 2). Using these group-averaged constraints, individual time-frequency windows were then created for each subject using their time-frequency plots created by averaging across all conditions (Figure 2).

Subject-specific time-frequency windows were used to estimate gamma and alpha power for illusion and non-illusion trials. For each individual, all time-frequency points were averaged within the previously defined, subject-specific gamma and alpha time-frequency windows so that a gamma and an alpha estimate were extracted for each condition for each individual. These values were then used in subsequent mixed ANOVAs and follow up *t*-tests with multiple comparison corrections to determine differences between illusion and non-illusion conditions and between groups. In addition, △ alpha (△ alpha = Illusion_Alpha_ – NonIllusion_Alpha_) and △ gamma (△ gamma = Illusion_Gamma_ – NonIllusion_Gamma_) values were quantified and used in an ANOVA with *post hoc* tests to understand the effect of group on the difference in alpha/gamma power between illusion and non-illusion trials. Because there were significant effects of group across all illusory SOA levels, illusory trials were combined within each group for statistical analysis. This statistical approach provides a non-biased, hypothesis-driven approach to determine the temporal and frequency range of interest at the group level while allowing for a wider range of frequencies across subjects, as is commonly observed in young versus older adults in the alpha range (Grandy et al., 2013). The subject-specific time-frequency windows increases sensitivity and accounts for individual differences across peak frequencies (Cohen, 2014).

In order to account for the lack of independence, avoid circular inference, and solve the issue of multiple comparisons, we followed a statistical approach similar to Hirano et al. (2018). We initially identified clusters of interest using unrestricted permutation mixed ANOVAs using the between-subjects factor, group (YA; NF; FH), and the within-subjects factor condition (Illusion; No Illusion) applied to PACz maps pooled across groups and conditions for 1,000 permutations (Manly, 2007). The estimated alpha value was the proportion of permuted *F* values that exceeded the original F statistic and clusters were defined using a threshold of *p* < 0.05 with *p* values adjusted by the positive False Discovery Rate (FDR) using the method outlined by Hirano et al. (2018).

Pearson correlations with a Bonferroni corrected alpha value of 0.005 (0.05/10) were conducted to examine the relationships between illusory strength and EEG measures (△ alpha, △ gamma, PACz) using R statistical software.

## Results

**FIGURE 3:**
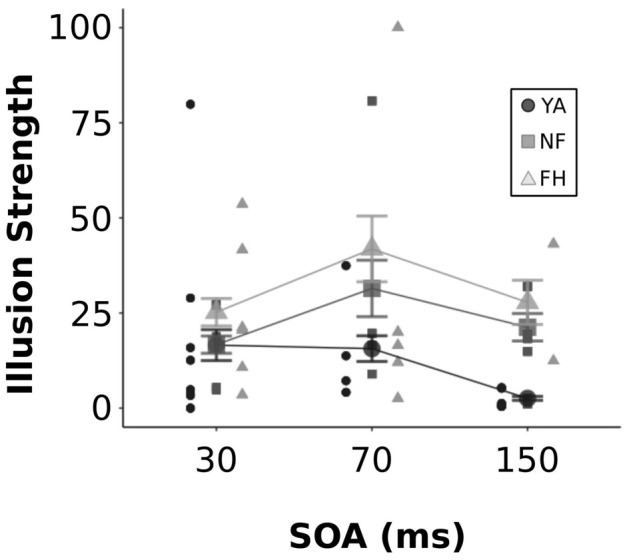
Illusion strength across SOA levels. Individual and group-averaged estimates of illusion strength are displayed for each SOA value. YA adults are represented by black circles, NF by dark gray squares, FH by light gray triangles. Error bars reflect standard error.

A two-way ANOVA was conducted on the perceptual data extracted from the SIFI EEG experiment (shown in Figure 3) to assess the significance of group and SOA on illusory strength. There was a significant effect of both group [*F*(2,183) = 11.98, *p* < 0.001] and SOA [*F*(2,183) = 4.52, *p* = 0.012] but no significant interaction [*F*(4,183) = 1.57, *p* = 0.18]. A *post hoc* Tukey HSD test showed that FH group had significantly greater illusion rates than both YA and NF (both adjusted *p* < 0.05) and NF had significantly greater illusion rates than YA (adjusted *p* = 0.037). A separate Tukey HSD test revealed significantly stronger illusion rates at the 70 ms SOA relative to the 150 ms SOA (adjusted *p* = 0.016) but illusory strength was not significantly different between 70 and 30 ms SOAs (adjusted *p* = 0.06) or between 150 and 30 ms SOAs (adjusted *p* = 0.74).

**FIGURE 4:**
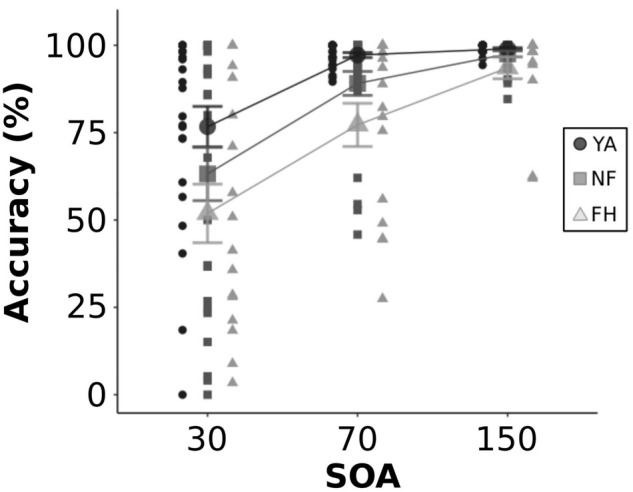
Accuracy during 2F2B congruent trials. Individual and group-averaged values for perceptual accuracy in discriminating 2 veridical flashes during congruent trials are displayed for each SOA level. YA adults are represented by black circles, NF by dark gray squares, FH by light gray triangles. Error bars reflect standard error.

In addition, we examined participant’s accuracy during 2F2B congruent trials (Figure 4). A mixed ANOVA was performed and revealed a significant effect of both group [*F*(2,60) = 5.13, *p* = 0.009] and SOA [*F*(2,120) = 54.13, *p* < 0.001] but no significant interaction [*F*(4,120) = 1.62, *p* = 0.17]. A *post hoc* Tukey HSD test revealed that there was no difference in accuracy between the NF group and the YA or the FH group (both adjusted *p* > 0.06) while the FH group did have significantly worse accuracy than the YA group (adjusted *p* < 0.001). Another *post hoc* Tukey HSD test showed that at both the 70 and 150 ms SOAs, accuracy was significantly better than the 30 ms SOA (both adjusted *p* < 0.001) while there was no significant difference in accuracy between the 70 and 150 ms SOAs (adjusted *p* = 0.10).

**FIGURE 5:**
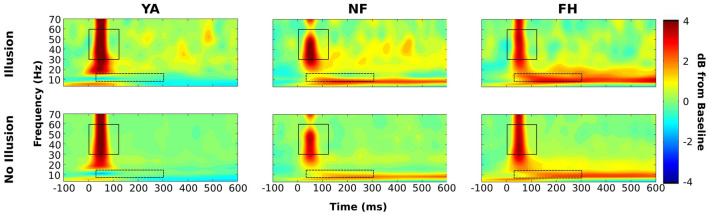
Time-frequency plots combined across SOA type. Group-averaged time-frequency representations are displayed for illusion (top row) and no illusion (bottom row) conditions combined across the 30, 70, and 150 ms SOA levels. Timepoint 0 ms represents the onset of the flash-beep pair and power spectra are displayed as the decibel (dB) change from the average –500 to –200 baseline period. Group level time frequency windows are shown for gamma (solid boxes) and alpha (dashed boxes). Power values are displayed in dB.

Next, we wanted to examine differences in alpha and gamma power between illusion and non-illusion trials (combined across SOAs) while participants performed the 1F2B illusion trials in the SIFI task. Time-frequency plots are shown in Figure 5 for each group and condition combined across SOA levels (Supplementary Figures 1–3 show time-frequency plots for each group and condition segregated by SOA level). Within occipital ROI, a mixed ANOVA was performed to examine the effect of group and condition (illusion versus non-illusion) on alpha power. There was a significant effect of group [*F*(2,34) = 180.7, *p* < 0.001] but not condition [*F*(1,34) = 0.04, *p* = 0.85] as well as a significant interaction [*F*(2,34) = 54.17, *p* < 0.001]. *Post hoc t*-tests with multiple comparisons (0.05/3 = 0.0167) were performed to examine the difference between condition within each group. There was no significant difference between conditions for YA adults [*t*(22) = 2.12, *uncorrected p* = 0.05] or for FH group [*t*(20) = 0.50, *uncorrected p* = 0.62] while NF group had significantly reduced alpha in illusion versus non-illusion conditions [*t*(26) = 10.50, *uncorrected p* < 0.001]. To further understand the significant interaction, we calculated the difference in alpha power between illusion and non-illusion trials (△ alpha = Illusion_Alpha_ – NonIllusion_Alpha_) for each individual and then conducted an ANOVA with a *post hoc* Tukey HSD test to understand the effect of group on this difference in alpha power. NF group had a significantly larger △ alpha compared to YA (adjusted *p* < 0.001) and compared to FH group (adjusted *p* < 0.001) but there was no difference in △ alpha between FH and YA (adjusted *p* = 0.66).

A similar analysis conducted for the gamma band demonstrated a significant effect of group [*F*(2,34) = 223.3, *p* < 0.001], of condition [*F*(1,34) = 6.96, *p* = 0.013], and a significant interaction [*F*(1,34) = 1479.24, *p* < 0.001]. Further *t*-tests adjusted for multiple comparisons revealed that the condition type did not induce significant differences in gamma power within YA [*t*(22) = 1.87, *uncorrected p* = 0.08] or NF group [*t*(26) = −2.2, *uncorrected p* = 0.04]. However, in FH group gamma power was significantly enhanced in illusory compared to non-illusory trials [*t*(20) = 6.22, *uncorrected p* < 0.001]. Again, we computed the difference in gamma power by subtracting non-illusion from illusion trials (△ gamma) for each individual and ran an ANOVA to examine the effect of group. The *post hoc* Tukey HSD test revealed that FH had a significantly larger △ gamma than NF and YA and that NF had a significantly larger △ gamma than YA (all adjusted *p* < 0.001).

**FIGURE 6:**
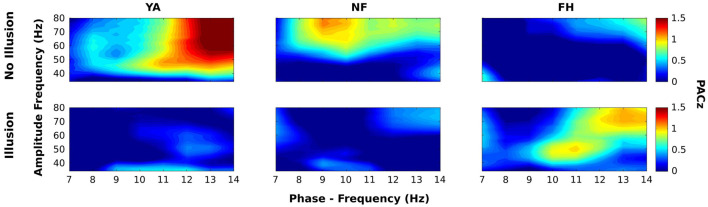
Group average maps of phase-amplitude coupling (PAC) within electrode Oz. Average PAC z scores across groups (YA: left column; NF: middle column; FH: right column) are displayed for each phase-amplitude pair during non-illusion (top row) and illusion (bottom row) conditions. For all maps, the phase frequency (7–14 Hz) is displayed along the *x*-axis while the amplitude frequency (30–80 Hz) is displayed along the *y*-axis.

**FIGURE 7:**
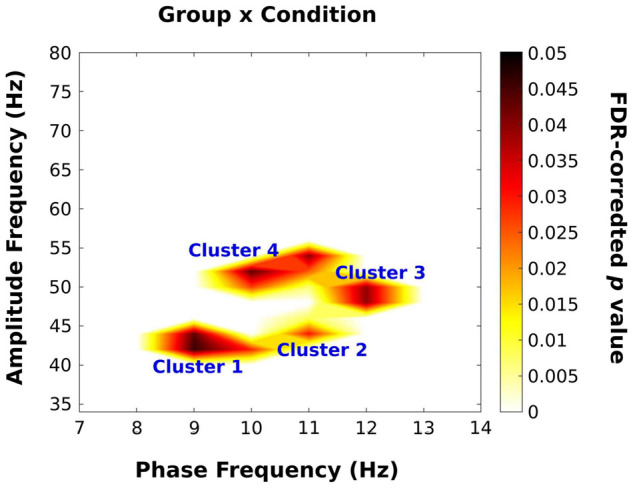
Results of permutation ANOVA analysis on PACz scores. A *p* value map with false discovery rate (FDR) corrected *ps* is displayed for the group × condition interaction. Any *p* value greater than 0.05 was converted to zero. There were 4 alpha/low-gamma clusters that revealed a significant interaction between group and condition. The main effects of group and condition did not retain any significant coupling following statistical analysis and are thus excluded.

Next, we examined the effect of trial type (illusory vs. non-illusory) and group on PACz. Figure 6 displays the PACz plots for each group (columns) across the non-illusion (top row) and illusion (bottom row) conditions. Significant clusters on these plots (shown in Figure 7) were identified by *F* values that exceeded those *F* values obtained from permutation ANOVAS (reported below) and had FDR-adjusted *p* values that passed a 0.05 threshold (see section “Materials and Methods”; Hirano et al., 2018). However, only the statistical map displaying the interaction [all *F*(2,28) > 3.43, all *p* < 0.047] is shown as the main effects of group and condition were not significant. As expected from the group PACz maps (Figure 6), this significant interaction occurred in Cluster 1 spanning a phase frequency between 8 and 10 Hz and an amplitude frequency between 40 and 46 Hz; Cluster 2 spanning a phase frequency between 10 and 12 Hz and an amplitude frequency between 42 and 48 Hz; Cluster 3 with a phase frequency ranging from 11 to 13 Hz and an amplitude frequency from 46 to 52 Hz; Cluster 4 with a phase frequency between 9 and 12 Hz and an amplitude frequency between 42 and 56 Hz. However, no statistically significant simple effects survived in follow up one-way ANOVAs that examined the effect of group on mean PACz in both non-illusion [all *F*(2,28) ≤2.23, all *p* >0.13] and illusion conditions [all *F*(2,28) ≤0.76, all *p* >0.48].

**FIGURE 8:**
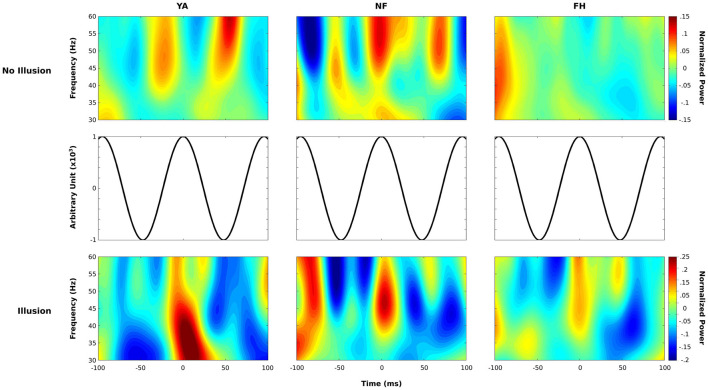
Phase-locked power spectrum of the gamma band. Normalized time-frequency maps are displayed for non-illusion (top row) and illusion (bottom row) conditions within each group (YA: left column; NF: middle column; FH: right column). Power estimates in the low gamma range were extracted within a 200 ms time window time-locked to the peak of 10.5 Hz alpha activity (middle row) and averaged over epochs. This methodology revealed bursts of gamma activity coupled to the troughs of alpha activity during no-illusion conditions in YA and NF group. Power values are displayed in dB.

To characterize the PAC within these clusters, time-frequency representations of the low-gamma band was aligned to the peaks of 10.5 Hz alpha activity. Figure 8 depicts the power spectra from the non-illusion condition (top row) and illusion condition (bottom row) aligned to the peaks of the alpha cycle (depicted in the middle row) in YA (left column), NF (middle column), and FH (right column). As is most evident in the YA group, bursts of gamma activity were aligned to troughs of the alpha band (∼ ± 50 ms) during non-illusion trials but to peaks of the alpha band (∼ 0 ms) during illusion trials. While the NF group demonstrates an equivalent pattern in both conditions, there is also gamma activity present during peak alpha activity in non-illusion trials (∼ 0 ms). Finally, the FH group doesn’t show substantial patterns of gamma activity during non-illusion conditions and altered pattern of gamma activity during illusion conditions with a burst around the alpha peak (∼0 ms) and at the alpha troughs (∼ ± 50 ms).

Finally, we investigated the correlations between the various electrophysiological measures (△ gamma,△ alpha, PACz) and the perceptual measure of illusion strength. In addition, we examined any correlation between age and these different variables. Although not significant at the Bonferroni corrected alpha value of 0.005 (0.05/10), weak relationships were found between illusion strength and PACz (*r* = −0.28, uncorrected *p* = 0.12) and between illusion strength and age (*r* = 0.33, uncorrected *p* = 0.02). There were not significant correlations between illusion strength and △ alpha (*r* = 0.15, uncorrected *p* = 0.39), between illusion strength and △ gamma (*r* = 0.01, uncorrected *p* = 0.94), between age and △ gamma (*r* = −0.09, uncorrected *p* = 0.61) or between age and △ alpha (*r* = 0.38, uncorrected *p* = 0.02). The full correlation matrix is reported in Table 3.

**TABLE 3:** Pearson r and associated uncorrected *p* values (in parentheses) from correlations between illusory strength and EEG measures (△ Alpha, △ Gamma, PACz) and age.

	**Illusory strength**	△ **Alpha**	△ **Gamma**	**PACz**	**Age**
**Illusory strength**	1.00	0.145 (.39)	0.013 (.94)	−0.282 (0.12)	0.33 (0.02)
△ **Alpha**		1.00	0.813 (<0.001)	−0.127 (0.50)	−0.38 (0.02)
△ **Gamma**			1.00	0.091 (0.63)	−0.09 (0.61)
**PACz**				1.00	0.11 (0.56)
**Age**					1.00

## Discussion

The purpose of this study was to examine differences in multisensory processing between YA, NF, and FH adults at both the perceptual and cortical levels. Prior studies have shown more severe impairments to multisensory temporal perception in older adults with a history of falls (Setti et al., 2011; Stapleton et al., 2014; Merriman et al., 2015) suggesting that more global cognitive deficits increasing an older individual’s susceptibility to a fall may also result in the deficits associated with multisensory processing. Using a combination of perceptual and EEG measures, the current findings help parse the specific differences in cortical processing during multisensory stimulation between the three groups.

Previous findings reporting more robust multisensory deficits in older adults with a fall history have used the SIFI task wherein increased susceptibility to the illusion reflects reduced precision in multisensory temporal perception. In line with prior findings, the current study found significant increases in illusion strength for the FH group compared to the YA and NF group and increased illusion strength for NF relative to YA group (McGovern et al., 2014; Stapleton et al., 2014; Merriman et al., 2015). It has been well established that natural aging results in reduced temporal precision and enhanced susceptibility to multisensory illusions (Setti et al., 2011; McGovern et al., 2014; Bedard and Barnett-Cowan, 2016; Baum and Stevenson, 2017; Scurry et al., 2019a,b). The present results show an exacerbated deficit in FH group, similar to prior findings (Setti et al., 2011; Merriman et al., 2015), possibly due to an altered ability of effective management of cortical resources during ambiguous or challenging information processing. Indeed, older fallers show significantly worse postural control than non-fallers when performing a challenging cognitive task while maintaining posture (Piirtola and Era, 2006; Stapleton et al., 2014). The increased illusion strength in FH may be due to central deficits in employing cognitive resources during the more difficult and conflicting illusory trials. In addition, degraded unisensory information is a normal consequence of the natural aging process leading to sensory reweighting (Ostroff et al., 2003; Humes et al., 2009; Werner et al., 2010; Alberts et al., 2019). This is enhanced in older adults prone to falls, often manifesting in greater reliance on visual cues (Jeka et al., 2010), which in the case of the SIFI may contribute to conflicting perceptual information and greater illusion susceptibility.

The FH group, but not NF, had significantly worse accuracy than YA in the congruent 2F2B trials. Along with sensory reweighting, differences in multisensory processing strategies may contribute to the impaired performance of FH. To compensate for age-related unisensory decline, older adults more heavily weight prior perceptual information in their predictive coding strategy, contributing to their increased illusion rate (Chan et al., 2021). This enhanced reliability on prior information is also seen in stronger rapid audiovisual recalibration effects in older compared to young adults, where the perception of audiovisual synchrony in a current trial is influenced by the temporal alignment of the preceding trial (Noel et al., 2016). As age-related declines in unisensory processing is enhanced in older fallers (Horak, 2006; Manor et al., 2010), it is plausible that FH group not only reweights sensory information but also relies more heavily on these internal perceptual templates leading to greater illusion rates. Future investigation of beta activity, thought to reflect predictive cortical processing (Bastos et al., 2012; Chan et al., 2021) may help pinpoint the contribution of perceptual priors in NF and FH groups processing and perception of the illusion versus non-illusion.

The current design used a single SOA for illusory trials but multiple SOAs for congruent trials. Recently, Chan et al. (2018) showed that both young and older adults perceived greater illusions with a reduced number of SOAs (3) compared to a larger number of SOAs (5). Presumably, more SOA levels provide additional information allowing for a more informed prediction strategy by the participant. Therefore, the single SOA used in the illusory trials may have affected illusion susceptibility across groups. Future studies manipulating this factor would be needed to understand this particular influence on FH versus NF and YA. Finally, a consistent and smaller step size in SOAs may allow for a better understanding of the temporal flexibility for the illusion in these different participant groups. For instance, prior studies show enhanced illusion susceptibility in older fallers at longer SOAs (110–270 ms w/40 ms step size) but not at shorter SOAs (30 and 70 ms) compared to older non-fallers and young adults (Setti et al., 2011). Setting the illusory SOA at the individual’s SIFI temporal threshold may be a more fruitful approach in future experiments to understand the temporal limits and the cortical mechanisms driving illusory vs. non-illusory percepts. This would also address the current limitation of having a small number of participants at the various illusory SOA levels by enhancing the statistical robustness of between group comparisons. In addition, this would reduce the likelihood of floor or ceiling performance by participants, a possible occurrence in the present study (i.e., some young adults had 0% susceptibility at 30 and 150 ms SOAs – see Figure 3).

In addition to altered illusory strength between groups, some interesting differences in gamma and alpha power within the current ROI were observed at the cortical level when contrasting illusion with non-illusion conditions. In the NF group only, alpha power was greater in non-illusion versus illusion trials and this △ alpha was significantly larger compared to FH and YA. However, when examining differences in gamma power, only the FH group demonstrated significantly greater gamma amplitude in the illusion compared to the non-illusion trials and this △ gamma was significantly larger compared to NF and YA, in line with our hypothesis.

In the young adults, the lack of any difference between illusory and non-illusory conditions in alpha and gamma power suggests they don’t require inhibitory control via alpha power modulating gamma power to detect the veridical single flash. More specifically, the low-level sensory processing, reflected in the gamma band, is dictated by temporal sensitivity and the population tuning profiles of early sensory areas (Meredith and Stein, 1983; Kayser et al., 2008; Schormans et al., 2017a). Therefore, young adults that demonstrate precise temporal sensitivity will process the physical visual signals accurately, without the need to filter or regulate this bottom up processing by higher-order controls, reflected in alpha activity (Bonnefond and Jensen, 2015).

In contrast, as shown in Figure 5, the NF group had a different pattern of gamma and alpha activity. The decreased alpha in illusion compared to non-illusion trials may indicate a faulty top-down mechanism that would lead to the enhanced illusion strength found in NF relative to YA. Increased gamma power with reduced alpha power has been specifically linked to the SIFI percept, presumably reflecting an increased readiness to integrate signals without proper top-down influence of signal processing (Bhattacharya et al., 2002; Cecere et al., 2015; for review see Keil, 2020). While there is a visible pattern of increased gamma activity for illusion compared to non-illusion (middle panel of Figure 5), this wasn’t significant limiting the current interpretation. Future studies that increase sample size, particularly for the PAC analysis, would be needed to identify any relationship between PACz and illusory strength and strengthen this interpretation. An increased sample size would also allow these correlations to be done separately within each group, possibly identifying a PACz-illusory strength relationship in some but not all groups furthering our understanding of this top-down control over SIFI percepts.

Finally, in the FH group, the significant increase in gamma power for illusion compared to non-illusion trials without concomitant modification in alpha power suggests a likely mechanism driving the increased susceptibility in this population. Enhanced low-level processing (i.e., increased gamma) of the sensory information without specific regulation by top-down processes (i.e., no change in alpha) would certainly lead to an illusory percept (Bhattacharya et al., 2002; Cecere et al., 2015; Keil and Senkowski, 2017). Synchronization of gamma activity across multiple networks increases feature integration and induces congruent multisensory percepts (Yuval-Greenberg and Deouell, 2007; Senkowski et al., 2008; Keil and Senkowski, 2018). Enhanced low-level sensory processing is consistent with what is known of neuronal responsiveness in sensory regions of aged animal models. For instance, the level of spontaneous activity is increased in the primary and secondary visual and auditory regions of aged animal cortices while tuning bandwidths become wider (Schmolesky et al., 2000; Leventhal et al., 2003; Fu et al., 2010; Gray et al., 2013; Ng and Recanzone, 2018). Therefore, the precision of bottom-up processing gets attenuated and top-down mechanisms would be required to filter out irrelevant sensory information from these initial processing stages. It should be noted that while typically alpha activity reflects top-down processes, as discussed, it can also represent bottom-up processing when generated from occipital region and feeding-forward to frontal areas (Wang et al., 2016). Therefore, the current interpretations based on changes in alpha power should be taken with some caution.

In addition, in the rat model of adult-onset hearing loss, firing rates were retained within multisensory and unisensory cortices, while the proportion of multisensory neurons decreased in the multisensory area but increased in primary auditory cortex (Schormans et al., 2017b). Therefore, it is likely that as age-related deterioration of sensory systems occurs, compensation ensues by altering the responsiveness of neurons in earlier, low-level stages of sensory processing. This theoretical framework would suggest that in the healthy older adult group (NF), increased alpha power is used to adjust for reductions in sensory processing precision. However, absence of such recruitment and increased low-level processing in the FH group suggests that this population suffers from global deficits in top-down control mechanisms enabling precision throughout the various functional, cognitive networks. In other words, FH group may suffer from deficits in top-down functionality (i.e., alpha activity) which is necessary to compensate for noisier and imprecise bottom-up processing (i.e., gamma activity).

Along with changes in the strength of oscillatory activity, coupling between lower frequency alpha and the higher frequency gamma has previously been shown to control sensory processing via “gating by inhibition” (Bonnefond and Jensen, 2015, 2013). As observed in Figure 6, YA and NF groups show an absence of PAC during illusion trials with more robust PAC in non-illusion trials. Presumably, sensory gating was present in YA (and to a lesser extent in NF) leading to a more accurate perception of the single, veridical flash. Curiously, the FH group shows weak PAC during illusion trials and a near absence during non-illusion trials, possibly contributing to the significantly worse accuracy during congruent trials.

Follow up analysis that aligned gamma power to peaks of alpha activity confirm that weak or absent PAC confers illusion susceptibility. In non-illusion trials, the bursts of gamma activity during troughs of the alpha cycle were clearly defined in the YA group while the NF group also exhibited gamma activity during the alpha peaks (Figure 8) suggesting a reduced, albeit relatively intact, capacity of sensory gating in this group. As preferred phase can influence the strength of PAC (Bonnefond and Jensen, 2015), the wider spread of gamma activity in NF (Figure 8) could help explain the weaker PAC found in this group (Figure 6). Regardless, the distinct shift in timing of gamma activity found in illusion compared to non-illusion trials in the YA groups suggests that gating by inhibition is a necessary mechanism that promotes veridical perception in young adults.

Indeed the alpha-phase locked power spectra analysis revealed that during the illusion percept, all three groups demonstrated gamma activity at peaks of the alpha cycle. This is expected as absence of sensory gating via the alpha band would lead to increased low-level sensory processing and subsequent perception of an illusory second flash. Interestingly, the FH group also showed some gamma activity during the troughs of the alpha cycle during the illusion condition which likely explain the PAC observed in this group, and not in YA or NF, during illusory trials (see Figure 6). However, the stronger increase in gamma activity found at the peak of the alpha cycle, and common across all three groups, likely drove the illusory percept. Overall, there appears to be a reduced capacity to effectively suppress processing of irrelevant or less reliable sensory information by the FH individuals as there was no robust pattern in PAC from illusion relative to non-illusion trials, unlike the clear distinctions found in the NF and YA groups.

The presented findings suggest that while perceptual measures of multisensory temporal processing are relatively intact in the FH group compared to NF, robust differences are present in the cortical processing driving these perceptual estimates. The increased gamma power in illusion trials for the FH group without any difference in alpha power in non-illusion trials implicate increased likelihood of multisensory integration, and thus the illusion, without proper top-down regulation of this bottom-up process. In contrast, the healthy NF group demonstrated increased alpha power in non-illusion compared to illusion trials indicative of more robust top-down gating on sensory processing enabling more precise and accurate perceptual representations. In addition, sensory gating by inhibition appears to be generally affected by the aging process as the NF group also exhibited weaker PAC and decreased suppression of gamma activity during peaks of the alpha cycle as compared to YA. Reduced PAC between alpha phase and gamma amplitude in non-illusion trials for the FH group suggest that top-down control of sensory processing is significantly impaired.

These various interpretations do need to be taken with some caution. Increasing the number of participants in the FH group to attain a balanced design would increase the statistical power and would likely improve the robustness of these preliminary findings. Further, the current experimental design that randomly assigned participants to a single SOA for illusory trials but multiple SOAs for congruent trials may have affected participant’s performance, as previously discussed. Future experiments can address these design issues and further examine how the number of SOAs and step-size of SOA may differentially affect FH from NF and YA groups. In addition, the potential contribution of predictive coding strategies may further explain the present results. Examining beta activity may show group differences and relationships with illusory rate confirming the proposed hypothesis of increased reliance on perceptual priors in FH and NF. Final limitations worth noting address the time-frequency and PAC analytical methods. The selection of group-level time-frequency windows could have induced experimenter bias and may benefit from future analyses that conduct random field test of time-frequency windows (Kilner et al., 2005). While wavelet transformation prior to extracting phase and amplitude is a common approach and shows enhanced PAC performance relative to other filtering methods (Caiola et al., 2019), bandpass filtering the data (i.e., wavelet transformations) can induce spurious phase-amplitude coupling and alternative methods may be useful in identifying PAC (Aru et al., 2015; Hülsemann et al., 2019; Munia and Aviyente, 2019). Nevertheless, the present results provide preliminary support for our hypothesis of a more drastic reduction in sensory gating via top-down inhibitory mechanisms in older adults with a history of falls.

## Data Availability Statement

The raw data supporting the conclusions of this article will be made available by the authors, without undue reservation.

## Ethics Statement

The studies involving human participants were reviewed and approved by the Institutional Review Board at the University of Nevada, Reno. The patients/participants provided their written informed consent to participate in this study.

## Author Contributions

AS and FJ designed the experiment. AS, ZL, and DL collected the data. AS performed data and statistical analysis with assistance on approach and interpretation from ZL, DL, and FJ. AS wrote the manuscript. FJ critically evaluated the manuscript. All authors contributed to the article and approved the submitted version.

## Conflict of Interest

The authors declare that the research was conducted in the absence of any commercial or financial relationships that could be construed as a potential conflict of interest.

## Publisher’s Note

All claims expressed in this article are solely those of the authors and do not necessarily represent those of their affiliated organizations, or those of the publisher, the editors and the reviewers. Any product that may be evaluated in this article, or claim that may be made by its manufacturer, is not guaranteed or endorsed by the publisher.
