# Supplementary material for: Impoverished Inhibitory Control Exacerbates Multisensory Impairments in Older Fallers
Source: Front Aging Neurosci. 2021 Sep 24;13:700787. doi: 10.3389/fnagi.2021.700787 (PMC8500399; doi:10.3389/fnagi.2021.700787)
Supplement: Supplementary file 1 [file Data_Sheet_1.docx]

Supplementary Material


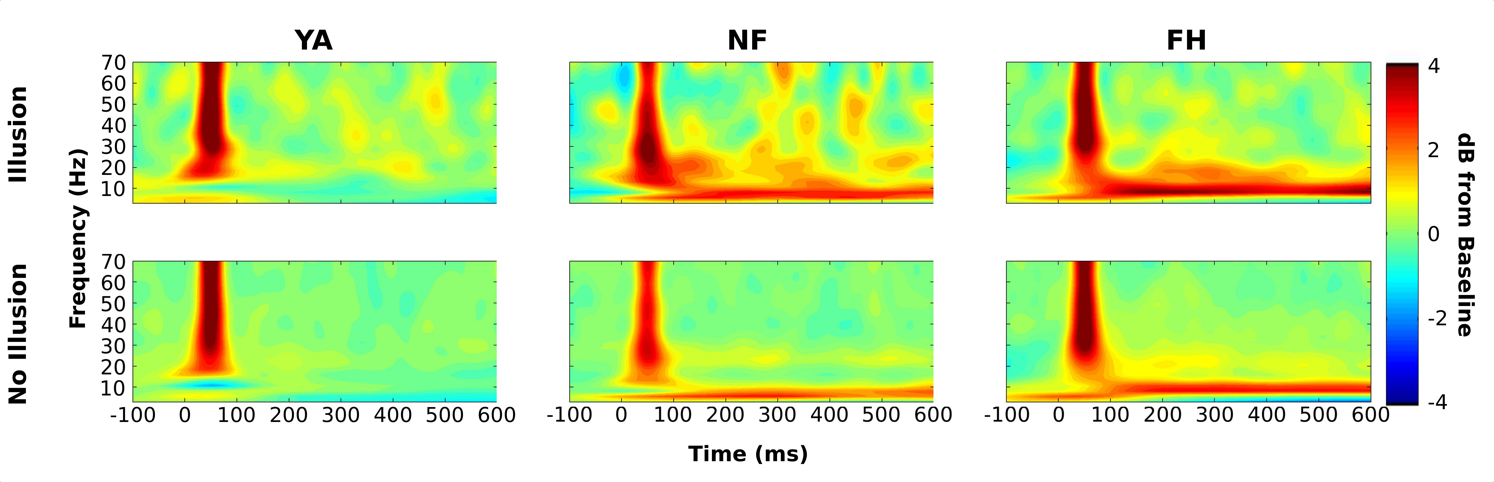
**Supplementary Figure 1.** Time-frequency plots for 30 ms SOA level. Group-averaged time-frequency plots are displayed for illusion (top row) and no illusion (bottom row) conditions during the 30 ms SOA level only.

**
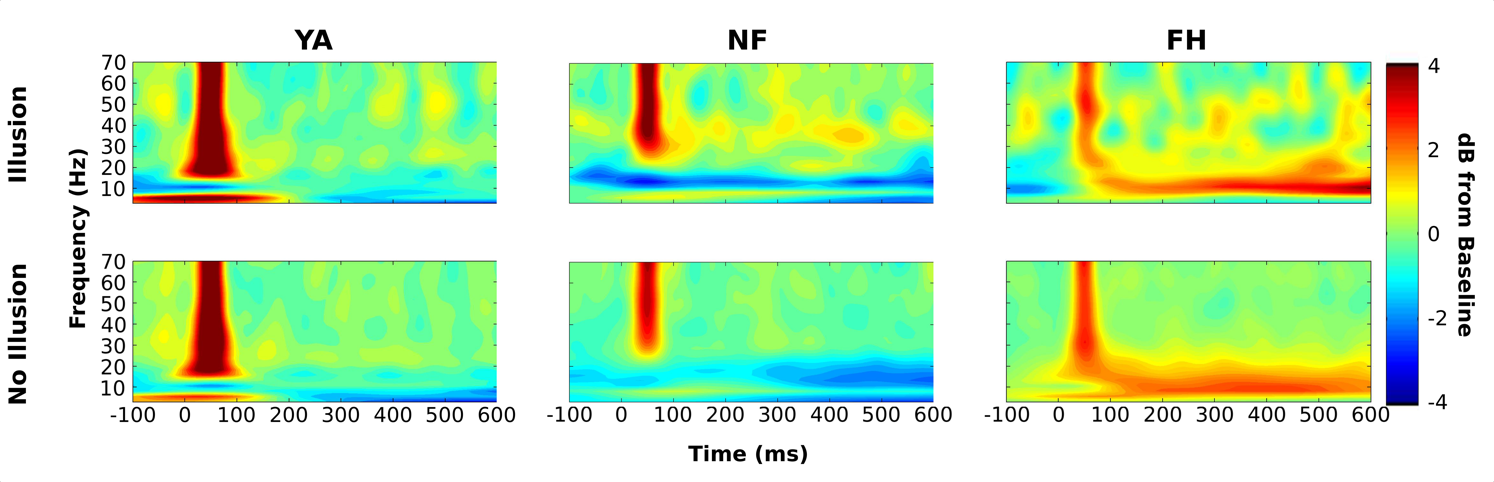
**

**Supplementary Figure 2.** Time-frequency plots for 70 ms SOA level. Group-averaged time-frequency plots are displayed for illusion (top row) and no illusion (bottom row) conditions during the 70 ms SOA level only.


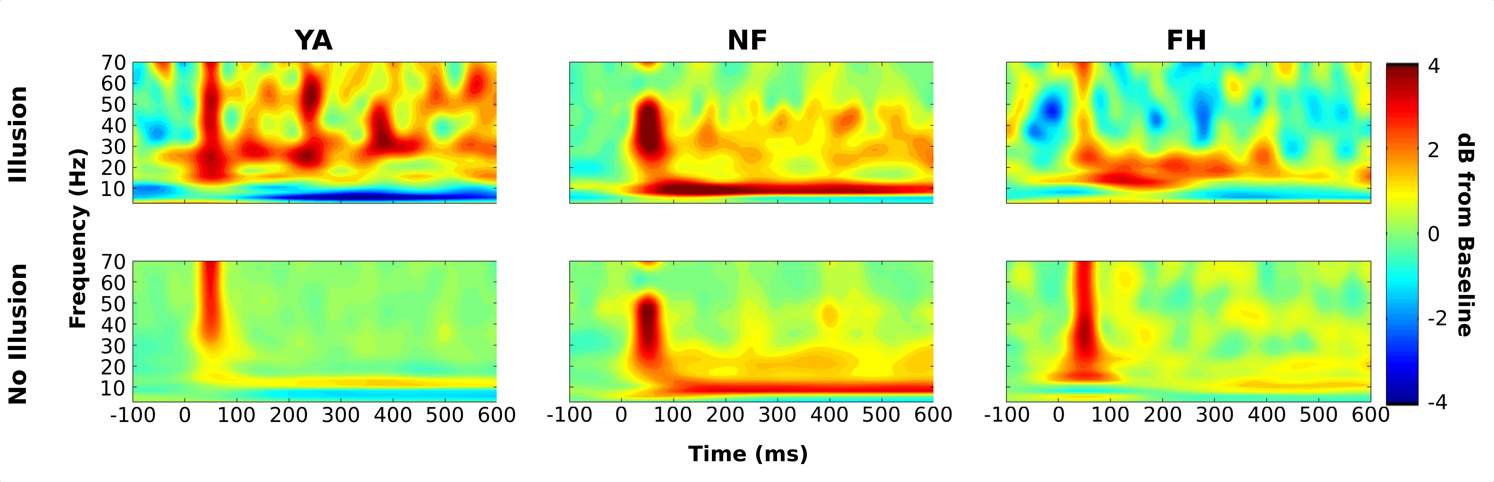


**Supplementary Figure 3.** Time-frequency plots for 150 ms SOA level. Group-averaged time-frequency plots are displayed for illusion (top row) and no illusion (bottom row) conditions during the 150 ms SOA level only.
